# Breaking Bad News: A Study on Formal Training in a High-Risk Obstetrics Setting

**DOI:** 10.1089/pmr.2020.0014

**Published:** 2020-05-28

**Authors:** Fernanda F. Oliveira, Glaucia R.G. Benute, Maria Augusta B. Gibelli, Nathalia B. Nascimento, Tercilia V.A. Barbosa, Renata Bolibio, Roberta C.A. Jesus, Paula V.V. Gaiolla, Maria Silvia V. Setubal, Ana L. Gomes, Rossana P. Francisco, Lisandra Stein Bernardes

**Affiliations:** ^1^ Disciplina de Obstetricia, Departamento de Obstetricia e Ginecologia, Faculdade de Medicina FMUSP, Universidade de Sao Paulo, Sao Paulo, Brazil.; ^2^ Grupo de Apoio Integral à gestantes e familiares de fetos com malformação (GAI), Divisao da Clínica Obstétrica, Hospital das Clinicas HCFMUSP, Faculdade de Medicina, Universidade de Sao Paulo, Sao Paulo, Brazil.; ^3^ Divisao de Pediatria, Hospital das Clinicas HCFMUSP, Faculdade de Medicina, Universidade de Sao Paulo, Sao Paulo, Brazil.; ^4^ Divisao de Enfermagem, Hospital das Clinicas HCFMUSP, Faculdade de Medicina, Universidade de Sao Paulo, Sao Paulo, Brazil.; ^5^ Divisão de Assistência Social, Hospital das Clinicas HCFMUSP, Faculdade de Medicina, Universidade de Sao Paulo, Sao Paulo, Brazil.; ^6^ Divisao de Psicologia, Hospital das Clinicas HCFMUSP, Faculdade de Medicina, Universidade de Sao Paulo, Sao Paulo, Brazil.; ^7^ Divisao de Cardiologia Pediátrica, Instituto do Coração INCOR, Hospital das Clinicas HCFMUSP, Faculdade de Medicina, Universidade de Sao Paulo, Sao Paulo, Brazil.; ^8^ Divisao de Clinica Obstetrica, Hospital das Clinicas HCFMUSP, Faculdade de Medicina, Universidade de Sao Paulo, Sao Paulo, Brazil.

**Keywords:** education, health communication, medical, obstetrics, simulation training

## Abstract

***Background:*** Breaking bad news is a frequent task in high-risk obstetrics clinics. Few studies have examined the role of training in improving such a difficult medical task.

***Aim:*** To evaluate the influence of a training program on the participants' perceptions of bad news communication at a high-risk obstetrics center.

***Design:*** This prospective study was conducted at the Department of Obstetrics/Gynecology, Hospital das Clinicas, from March 2016 to May 2017.

***Setting/Participants:*** Maternal-fetal health specialists were invited to complete an institutional questionnaire based on the SPIKES protocol for communicating bad news before and after training. The training consisted of theoretical lectures and small group practice using role play. The questionnaire responses were compared using nonparametric tests to evaluate the differences in physicians' perceptions at the two timepoints. The questionnaire items were evaluated individually and in groups following the communication steps of the SPIKES protocol.

***Results:*** In total, 110 physicians were invited to participate. Ninety completed the pretraining questionnaire and 40 answered the post-training questionnaire. After training, there were significant improvements in knowing how to prepare the environment before delivering bad news (*p* = 0.010), feeling able to transmit bad news (*p* < 0.001), and to discuss the prognosis (*p* = 0.026), feeling capable of discussing ending the pregnancy (*p* = 0.003), and end-of-life issues (*p* = 0.007) and feeling confident about answering difficult questions (*p* = 0.004). The comparison of the grouped responses following the steps of the SPIKES protocol showed significant differences for “knowledge” (*p* < 0.001), “emotions,” (*p* = 0.004) and “strategy and summary” (*p* = 0.002).

***Conclusion:*** The implementation of institutional training in breaking bad news changed the perception of the physicians in the communication setting.

## Background

Technological development and diagnostic advances have allowed for detection of early fetal abnormalities in the first months of pregnancy.^1–3^ However, although such advances have enabled changes in some pathologies and facilitated the offer of hope to patients, they have also created a need for greater medical skills in delivering and discussing bad news while the fetus is still *in utero*.^1–3^

Bad news can be defined as any information that negatively changes expectations about the future.^1,2^ In obstetrics, bad news may be related to any event during pregnancy that leads to some risk to maternal and fetal health and breaks parental expectations.^3–6^ In this way, breaking bad news in the area of obstetrics is painful and characterized by the experience of many emotions, both for the family involved and for the professional transmitting the bad news.^1,3–6^

Studies described in oncology report that the way news is transmitted directly affects a patient's understanding, contentment with medical care, the level of hope, and subsequent short- and long-term psychological adaptation.^7,8^ The literature in this area also demonstrates that, just as it impacts the patient's life, the communication of bad news generates a situation of extreme stress for the health team member.^1,5,7–13^ The development of specific professional training projects in bad news communication has shown, especially in oncology and geriatrics, that communication skills can be improved in both the professionals' and the patients' perception.^1,5,7–13^ Similar to the results described in oncology and geriatrics literature, qualitative studies in the obstetrics settings have shown that the way bad news is transmitted directly influences parental reactions.^3,14^ However, few studies propose some type of professional training in breaking bad news. Considering that maternal–fetal medicine centers provide high-risk maternal and fetal care, where adverse diagnoses are realized many times, it is important to study the training that is offered in this setting. Therefore, the aim of our study was to evaluate the impact of institutional training on physicians' perceptions of breaking bad news.

## Aims

The aim of the study was to evaluate the impact of training on physicians' perceptions of communicating bad news.

## Materials and Methods

### Design

A prospective study was conducted at the Department of Obstetrics and Gynecology, Hospital das Clinicas (HC), Faculdade de Medicina FMUSP, Universidade de São Paulo, from March 2016 to May 2017. The Obstetrics Department of the HC-FMUSP is responsible for 2200 prenatal appointments a month in the city of São Paulo, Brazil. Of the patients who attend these appointments, 90% have high maternal–fetal risk, defined by the presence of any clinical or obstetric condition pre-existing or developed during the prenatal period, which confers a real or potential risk to the health or well-being of the mother or fetus.^15^

### Setting

One hundred eleven physicians who work in the Obstetrics Department of the HC-FMUSP were invited to participate in the study.

To evaluate the physicians' perceptions of breaking bad news before and after institutional training, an institutional questionnaire was applied twice: once before the training and once after the training. All questionnaires that were answered by a physician were included in the analysis. The procedure and training are specified hereunder.

### Data collection

Data were collected from anonymous institutional questionnaires administered immediately before the beginning of the proposed training and about three months after the training. The questionnaire was administered to the entire medical staff of the obstetrics clinic. All the physicians who answered the questionnaire before the training were part of the cohort labeled Step 1. All the physicians who answered the questionnaire after the complete training were part of the cohort labeled Step 2.

To involve the most physicians, the complete training (theoretical and practical) was performed at three different times. The interval between administration of the first questionnaire (before training) and the application of the last questionnaire was 14 months.

#### Institutional questionnaire (*Questionnaire for the analysis of perceptions of breaking bad news specific to the obstetric area*)

The questionnaire was formulated based on a review of the existing literature on breaking bad news and protocols used for it and aimed to identify the main perceptions and attitudes of professionals regarding breaking bad news.

After the questionnaire was developed, a multidisciplinary meeting was held to analyze the issues with seven professionals from different areas (obstetrics, nursing, social assistance, and psychology) working in our perinatal palliative care team to clarify the questions and modify those with potentially unclear meanings.

The final questionnaire is provided in Supplementary Data S1 and its English translation is provided in Supplementary Data S2.

The final questionnaire for the analysis of the perceptions of breaking bad news specific to the obstetric area (QAPBBN-O) was composed of 13 demographic questions, 2 open-ended questions, and 24 affirmations using a Likert scale that was created based on the SPIKES protocol.

#### Training

The training model proposed in this study consisted of two stages: theoretical training and practical training using simulation.

##### Theoretical training

The theoretical training consisted of a lecture offered for all health professionals working in the obstetrics department.

The lecture had a mean duration of 50 minutes and presented the following topics: a brief definition of bad news, examples of bad news communications in the obstetrics setting, studies on perceptions of patients receiving bad news in perinatology scenarios, and a communication protocol based on the SPIKES protocol adapted to the obstetrics setting.^1,2,5,14,16,17^

##### Practical training with simulations

The professionals were organized into groups of a maximum of 12 people per shift. The duration of the practical training sessions was ∼90 minutes. A moderator trained to conduct the training led all sessions (L.S.B. or F.F.O.).

Two fictitious scenarios based on real cases were created by the authors (L.S.B. and F.F.O.) (Supplementary Data S3). The participants were asked to voluntarily play the role of the patient, the relative, or the health professional in the scenarios. A debriefing was carried out after the scenarios were acted out. During the debriefing, the participants were encouraged to describe their feelings about breaking the bad news (if they played the role of the physician) or how they felt when they received the bad news (if they had played the role of the patient or relative). The participants were also encouraged to give feedback about the training.^9,18–26^

#### Practical training feedback questionnaire

Immediately after the practical training, the participants were invited to complete a feedback questionnaire; for each question, a response scale ranging from extremely little (0) to extremely (10) was used. This questionnaire is provided in Supplementary Data S4 and its English translation is provided in Supplementary Data S5.

#### Procedure

The training and data collection consisted of five different components:

1. Application of the baseline questionnaire *(QAPBBN-O-S1)*
2. Theoretical training
3. Practical training with simulations
4. Application of the practical training feedback questionnaire
5. Application of the follow-up questionnaire (*QAPBBN-O-S2)*

**FIG. 1.**
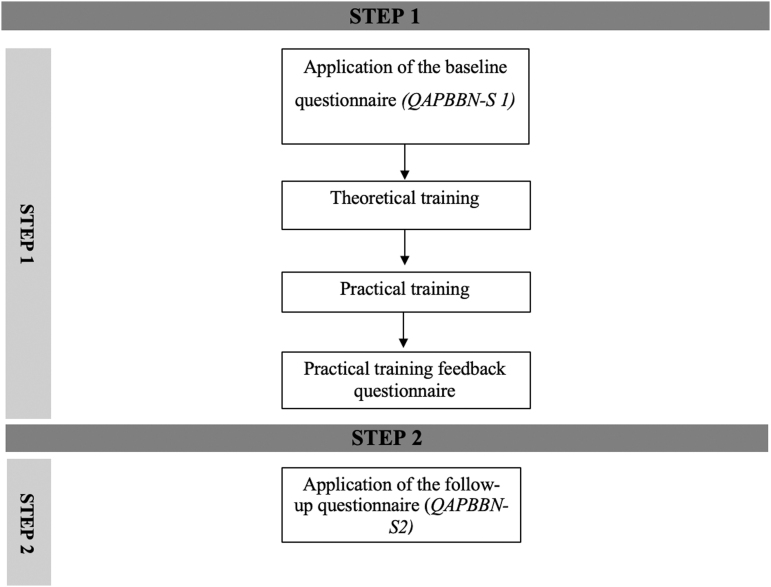
Fluxogram—breaking bad news training steps and institutional questionnaire application.

The flowchart demonstrating the steps of the proposed training and the moments of application of the questionnaires is shown in Figure 1.

The IBM SPSS program, version 20.0, was used for data storage and statistical analysis.

The quantitative variables are presented as the mean, standard deviation, median, minimum, and maximum. The qualitative variables are presented as absolute and relative frequencies (%).

The difference in the Likert scale points before and after the training was tested. In addition, to evaluate the steps of the *SPIKES* protocol, the statements were grouped according to each step (“setting up,” “perception,” “invitation,” “knowledge,” “emotions,” “strategy and summary”), and the sum of scores obtained from the Likert scales was calculated for each step. These group scores were called the *SPIKES* scores.

The Kolmogorov–Smirnov test was used to test the normality of the quantitative data. The qualitative variables were compared using the Mann–Whitney U test for two independent samples. Chi-square or Fisher's exact tests were also used to evaluate the agreement of the categorical variables.

#### Sample size

A convenience sample of all 111 physicians in the department who completed the questionnaires was used.

#### Ethical approval

The project was approved by the Ethics and Research Committee of the HC-FMUSP in 2016 March 30 with number 54697216.0.00000.0068.

## Results

**FIG. 2.**
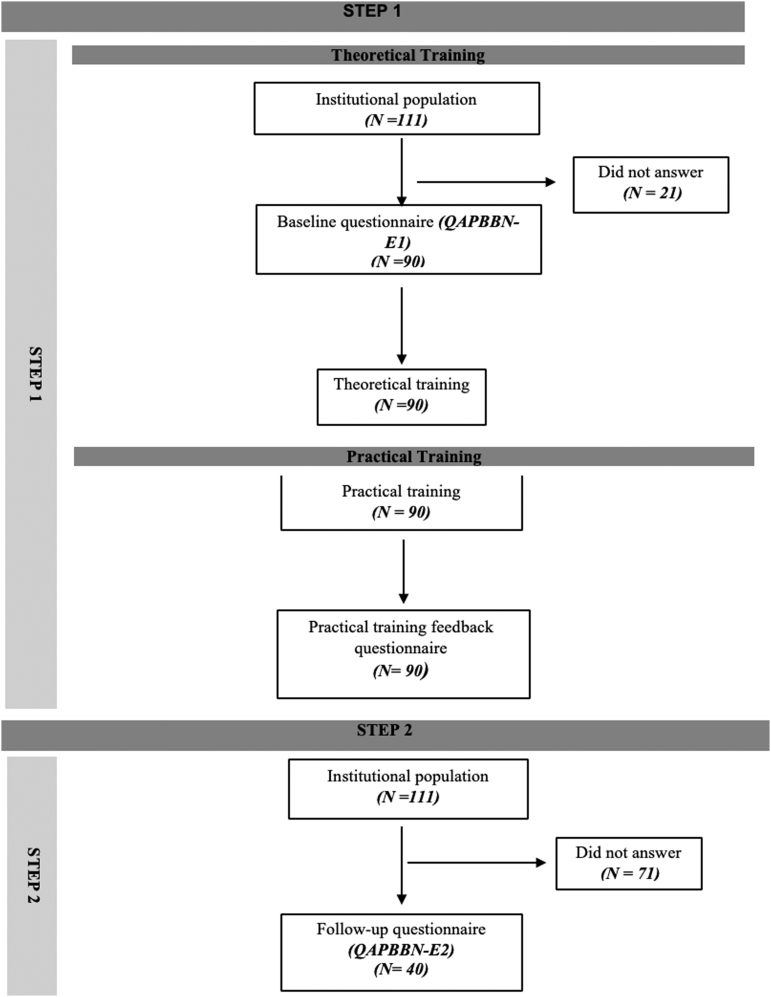
Fluxogram—physicians' participation in the training process.

A total of 111 physicians were invited to participate in the training. Ninety (81.1%) answered the initial questionnaire *(QPABBN-O-E1)* and participated in the theoretical training. These 90 physicians were distributed into 11 groups for practical training, and all of them completed the practical training feedback questionnaire. Forty physicians (36%) answered the final questionnaire *(QAPBBN-O-E2)*. The interval between the first application of the questionnaire and the training (theoretical and practical) of the entire team until the last application of the questionnaire was 14 months. The attrition rate for the obstetrics clinical staff during the research was 16.1%. A flowchart explaining the participation of the physicians at each step is shown in Figure 2. To evaluate the possible differences between the population in Step 1 and Step 2, the demographic data for the two groups were compared (Table 1). There was no significant difference in the baseline characteristics between the groups.

**Table 1.** Comparison of the Demographic Characteristics between the Groups

Demographic variables	Mean ± SD/*n* (%)		*t*-Test/chi-square
	Step 1	Step 2	
Age (years)	33.5 ± 1.2	36.8 ± 2.1	0.187
Years from graduation	8.9 ± 1.2	12.2 ± 2.0	0.157
Female	71 (80)	30 (75)	0.546
Has children	20 (22.5)	28 (30)	0.365
Follows a religion	76 (85)	38 (95)	0.145
Expertise Obstetrics/gynecology	76 (84.4)	38 (95)	0.250
Expertise fetal medicine	14 (15.6)	2 (5)	

SD, standard deviation.

### Analysis of the practical training feedback questionnaire

The description of the analysis of the practical training feedback questionnaire is given in Table 2. The answers were evaluated on a scale of 0 to 10.

**Table 2.** Analysis of the Practical Training Feedback Questionnaire Responses of the 90 Physicians Who Participated in the Training

Questions	Mean ± SD	Median (min.–max.)
*How much is communication a part of your life?*	7.6 ± 2.1	8 (3–10)
*How difficult does it feel to break bad news?*	7.3 ± 2.0	8 (0–10)
*How much time in your training or professional qualification was dedicated to communication attention?*	2.1 ± 1.5	2 (0–7)
*To what extent did you think you were prepared to break bad news before the role play?*	5.5 ± 2.1^a^	5 (1–10)
*To what extent do you feel prepared to break bad news after the role play?*	7.7 ± 1.2^a^	8 (4–10)
*How do the situations staged in role play simulate a real case?*	8.9 ± 1.5	8 (3–10)
*How useful was the role play?*	8.9 ± 1.7	10 (1–10)

^a^ The analysis of repeated measures between the perception of being better prepared to break bad news before and after role play is statistically significant (*p* < 0.001).

As we can observe, the transmission of bad news is part of the participants' daily life (median 8/10) and is considered a difficult task in clinical practice (median 8/10). The answers also indicated that there is no specific technical training dedicated to this type of communication during medical training (median 2/10). In addition, the participants positively valued the proposed training model (median 10/10) and perceived that they were better prepared to break bad news after the training (a median of 5/10 before training and 8/10 after training). The difference in the participants' perceptions of their preparation to communicate bad news before and after the practical training was statistically significant (*p* < 0.001).

### Analysis of the questionnaires

The comparison of the affirmations and the *SPIKES* scores between the two steps is described in Supplementary Table S1 and Table 3.

**Table 3.** Effect of Training on the *SPIKES* Scores

SPIKES score	Median (min–max)		p
	Step 1	Step 2	
“Setting up” [Preparation of the professional and the environment in which the news will be transmitted]	31 (11–35)	32.5 (23–35)	0.050
“Perception” [Assessment of the extent to which the patient is aware of his/her condition]	5 (2–5)	5 (2–5)	0.255
“Invitation” [Development of an understanding of how much the patient wants to know about his/her illness]	8 (3–10)	8.5 (4–10)	0.534
“Knowledge” [Transmission of information to the patients and evaluation of the impact of transmitting bad news]	19 (7–30)	23.5 (10–26)	**<0.001**
“Emotions” [Response to the patient's reaction]	3 (1–5)	4 (1–5)	**0.004**
“Strategy and summary” [Disclosure of therapeutic plan and perinatal palliative care]	8 (3–8)	10 (7–11)	**0.001**

Bold values indicate values statistically significant differences between steps, *p* < 0.05.

The main affirmations that were different before and after the training were “If I am performing an ultrasonography examination, I turn on the lights to report bad news” (*p* = 0.010); “I feel comfortable breaking bad news” (*p* = 0.013); “I feel prepared to break bad news” (*p* < 0.001); “I have the ability to break bad news” (*p* < 0.001); “I have the ability to discuss the prognosis” (*p* = 0.012); “I feel confident answering difficult questions asked by patients during the communication of bad news” (*p* = 0.004); “I am skilled in talking about the end of pregnancy or the beginning of palliative care” (*p* = 0.003); and “I am skilled in discussing issues related to the end of life” (*p* = 0.007).

As shown, there was a significant difference between the groups before and after training for the variables “knowledge” (*p* < 0.001), “emotions” (*p* = 0.004), and “strategy and summary” (*p* = 0.002).

## Discussion

### Breaking bad news training in obstetrics settings

Our study demonstrates for the first time that formal training on delivering bad news in a tertiary obstetrics center impacts physicians' overall perceptions of their communication skills, including their confidence to perform such a task. In addition, the analysis shows that very little time had been dedicated to communication training during the physicians' medical training and beyond. Institutional training is desirable and may impact patients' long-term recovery after stressful events such as the loss of a child during pregnancy or the diagnosis of a life-limiting fetal condition.

In the past few years, many publications on breaking bad news have analyzed communication patterns among health professionals, the barriers to transmission of bad news, and the impact of the communication of bad news on patients, showing the lack of formal training during professional studies and the need to implement such training in other specialties, such as oncology.^2,7,8,10,11^ In the context of perinatology, few studies have evaluated the implementation of formal training, and no study has evaluated institutional training in everyday obstetrics care.^27–30^

In the context of neonatal care, few authors have described the process of training in breaking bad news. In 2014, Tobler et al.^27^ conducted a study to evaluate the efficacy of simulated training in communicating bad news among pediatric residents. In the study, the authors conducted theoretical training that was associated with a simulated situation and was followed by a debriefing. The study included 39 physicians and trainees in pediatrics. At the end of the training, the authors concluded that there were improvements in the participants' ability to transmit bad news.^27^ Similar results were found in 2015 by Marko et al.,^28^ who, through a study involving 77 medical students, noted that structured curriculum implementation improved the students' performance of patient counseling regarding bad news and that students had increased levels of confidence and empathy.^28^ Also in the area of perinatology, in 2016 Karkowshy et al.^29^ used a combination of theoretical training, simulated practice, and debriefing to administer training to physicians in gynecology and obstetrics and found improvements in the participants' perceptions of their capacity to break bad news and in their verbal and nonverbal communication. The same authors later reported long-term improvement in the participants' communication capacity.^29^ More recently, in 2018, Setubal et al.^30^ conducted a study with 61 physician trainees in perinatology and demonstrated an increase in their performance scores in the communication of bad news; in addition, the authors found that simulated training was valued by the participants.^30^

Physicians' difficulty in dealing with patients' emotions and their own feelings of helplessness when the success of treatment is unlikely and expectations are not met are key factors in the communication of bad news being delayed or inappropriately delivered.^1,3–5,31^ The implementation of formal training provides a tool for participants to be able to deal with these issues, supporting their perceptions of a greater capacity to perform this function.^29,32–36^

### Strengths and limitations

The results of this study should be considered in light of several strengths and limitations. Although most physicians (81.1%) in the department participated in the theoretical and practical training, some were not present for personal reasons. Also, although baseline characteristics were similar in Step 1 and Step 2 questionnaires, the frequency of responders of Step 2 questionnaires was 40%. As the application of the questionnaire was institutional and anonymous, it is not possible to ensure that all participants in the second stage participated in the training. However, the aim of the study was to evaluate how physicians at the institution reacted to the formal training. The participants who did not complete the formal training were most likely aware of the communication tools and their impact on the institutional culture concerning how bad news is communicated, as described in the literature about organizational learning.^37,38^

### Main findings

At the end of our study, we believed that the institutional approach was able to increase the importance placed on the subject of bad news communication. Despite this observation of changes in the institutional culture in relation to the communication of bad news, further evaluations are still necessary to verify whether these changes are sustained in the long term.

## Conclusion

The institutional formal training had a positive impact on the perceptions of the involved health professionals in the department. Further studies are needed to assess whether this institutional training is able to create long-term changes in the institutional culture.

## Supplementary Material

Supplemental data

## Abbreviation Used

SD: standard deviation
