# Supplementary material for: Breaking Bad News: A Study on Formal Training in a High-Risk Obstetrics Setting
Source: Palliat Med Rep. 2020 May 28;1(1):50–7. doi: 10.1089/pmr.2020.0014 (PMC8241325; doi:10.1089/pmr.2020.0014)
Supplement: Supplemental data [file Suppl_Data.pdf]

Supplementary Data

Supplementary Data S1. Original Questionnaire (Portuguese Language)

Questionário de percepção em comunicação de más-notícias em obstetrícia (QPCMN-O)

Dados pessoais

1-Qual sua profissão?
2-Qual sua área de atuação?
3-Você faz parte de qual equipe?
4-Qual sua idade?
5-Quantos anos tem de formado?
6-Qual seu sexo?
7-Você tem filhos?
8-Já teve algum bebe com malformação ou óbito fetal?
9-Se a resposta for positive, qual era o sexo do bebê?
10-Segue alguma religião?
11-Se a resposta for positive, qual sua religião?
12-Você participa de sua comunidade religiosa?
13-Se a resposta for positive, quantas vezes por mês??

Questão aberta:

1-O que você considera uma má notícia??
2-No ultimo mês, quantas vezes você comunicou uma má notícia?

Afirmações utilizando escala Likert:

Por favor responda o questionário abaixo sobre a forma como você usualmente comunica uma má-notícia:

A1. "Se estou fazendo o exame, acendo as luzes para comunicar a má notícia"

( ) Discordo totalmente
( ) Discordo parcialmente
( ) Não discordo nem concordo
( ) Concordo parcialmente
( ) Concordo totalmente

A2. "Peço para a gestante se sentar antes de comunicar a má notícia"

( ) Discordo totalmente
( ) Discordo parcialmente
( ) Não discordo nem concordo
( ) Concordo parcialmente
( ) Concordo totalmente

A3. "Peço para gestante se vestir antes de comunicar a má notícia"

( ) Discordo totalmente
( ) Discordo parcialmente
( ) Não discordo nem concordo
( ) Concordo parcialmente
( ) Concordo totalmente

A4. "Levo a gestante para outra sala para comunicar a má notícia"

( ) Discordo totalmente
( ) Discordo parcialmente
( ) Não discordo nem concordo
( ) Concordo parcialmente
( ) Concordo totalmente

A5. "Considero ser importante a privacidade do ambiente em que se dá a má notícia."

( ) Discordo totalmente
( ) Discordo parcialmente
( ) Não discordo nem concordo
( ) Concordo parcialmente
( ) Concordo totalmente

A6. "Se a gestante estiver realizando algum exame sem acompanhante solicito que este esteja presente ao comunicar a má notícia"

( ) Discordo totalmente
( ) Discordo parcialmente
( ) Não discordo nem concordo
( ) Concordo parcialmente
( ) Concordo totalmente

A7. "Quando comunico uma má notícia, penso antes como irei falar"

( ) Discordo totalmente
( ) Discordo parcialmente
( ) Não discordo nem concordo
( ) Concordo parcialmente
( ) Concordo totalmente

A8. “Me sinto confortável ao comunicar a má notícia”

( ) Discordo totalmente
( ) Discordo parcialmente
( ) Não discordo nem concordo
( ) Concordo parcialmente
( ) Concordo totalmente

A9. “Me sinto calmo ao comunicar a má notícia”

( ) Discordo totalmente
( ) Discordo parcialmente
( ) Não discordo nem concordo
( ) Concordo parcialmente
( ) Concordo totalmente

A10. “Me sinto preparado para comunicar a má notícia”

( ) Discordo totalmente
( ) Discordo parcialmente
( ) Não discordo nem concordo
( ) Concordo parcialmente
( ) Concordo totalmente

A11. “Tenho habilidade para transmitir a má notícia”

( ) Discordo totalmente
( ) Discordo parcialmente
( ) Não discordo nem concordo
( ) Concordo parcialmente
( ) Concordo totalmente

A12. “Antes de comunicar a notícia pergunto ao paciente o que ele já sabe sobre o exame ou a situação sobre a qual vamos conversar”

( ) Discordo totalmente
( ) Discordo parcialmente
( ) Não discordo nem concordo
( ) Concordo parcialmente
( ) Concordo totalmente

A13. “Tenho habilidade em dar espaço para gestante falar.”

( ) Discordo totalmente
( ) Discordo parcialmente
( ) Não discordo nem concordo
( ) Concordo parcialmente
( ) Concordo totalmente

A14. “Procuro saber o quanto de informações o paciente quer antes de conversar sobre a notícia.”

( ) Discordo totalmente
( ) Discordo parcialmente
( ) Não discordo nem concordo
( ) Concordo parcialmente
( ) Concordo totalmente

A15. “Tenho habilidade em discutir sobre o diagnóstico”

( ) Discordo totalmente
( ) Discordo parcialmente
( ) Não discordo nem concordo
( ) Concordo parcialmente
( ) Concordo totalmente

A16. “Tenho habilidade em falar sobre o prognóstico”

( ) Discordo totalmente
( ) Discordo parcialmente
( ) Não discordo nem concordo
( ) Concordo parcialmente
( ) Concordo totalmente

A17. “Tenho habilidade em falar sobre o fim da gravidez ou início de tratamento paliativo”

( ) Discordo totalmente
( ) Discordo parcialmente
( ) Não discordo nem concordo
( ) Concordo parcialmente
( ) Concordo totalmente

A18. “Tenho habilidade em discutir questões relativas ao fim da vida”

( ) Discordo totalmente
( ) Discordo parcialmente
( ) Não discordo nem concordo
( ) Concordo parcialmente
( ) Concordo totalmente

A19. “Me sinto seguro para responder perguntas difíceis formuladas por pacientes durante a revelação da má notícia.”

( ) Discordo totalmente
( ) Discordo parcialmente
( ) Não discordo nem concordo

( ) Concordo parcialmente
( ) Concordo totalmente

A20. "Tive treinamento ou ensinamento específico para comunicação de má notícia."

( ) Discordo totalmente
( ) Discordo parcialmente
( ) Não discordo nem concordo
( ) Concordo parcialmente
( ) Concordo totalmente

A21. "Considero importante a utilização de uma estratégia ou protocolo para comunicar uma má notícia"

( ) Discordo totalmente
( ) Discordo parcialmente
( ) Não discordo nem concordo
( ) Concordo parcialmente
( ) Concordo totalmente

A22. "Tenho conhecimento de algum protocolo de comunicação de más-notícias"

( ) Discordo totalmente
( ) Discordo parcialmente
( ) Não discordo nem concordo
( ) Concordo parcialmente
( ) Concordo totalmente

CASO VOCE JA TENHA TIDO CONTATO COM ALGUM PROTOCOLO RESPONDA AS QUESTOES SEGUINTES:

A23. "Apesar de conhecer algum protocolo de comunicação de más-notícias prefiro utilizar a prática clínica para comunicar más-notícias"

( ) Discordo totalmente
( ) Discordo parcialmente
( ) Não discordo nem concordo
( ) Concordo parcialmente
( ) Concordo totalmente

A24. "Considero que o uso desse protocolo ajuda na prática clínica."

( ) Discordo totalmente
( ) Discordo parcialmente
( ) Não discordo nem concordo
( ) Concordo parcialmente
( ) Concordo totalmente

Pergunta aberta

POR FAVOR DESCREVA SUCINTAMENTE O PROTOCOLO DE COMUNICAÇÃO DE MÁS-NOTÍCIAS QUE UTILIZA.

Supplementary Data S2. Original Questionnaire (English Translation)

Questionnaire for the analysis of perceptions of breaking bad news specific to the obstetric area *(QAPBBN-O)*

Personal data

1-What is your occupation?
2-What is your area of professional activity?
3-Which health team are you a part of?
4-How old are you?
5-How many years ago did you graduate?
6-What is your gender?
7-Do you have kids?
8-Have you ever had a baby with a malformation or experienced fetal death?
9-If the answer was yes, what was the baby's gender?
10-Are you religious?
11-If the answer was yes, what is your religion?
12-Do you participate in your religious community?
13-If the answer was yes, how many times per month?

Open-ended questions

1-What is considered bad news?
2-In the last month, how many times did you break bad news?

Affirmations using a Likert scale

Please answer the questionnaire below about how do you usually break bad news:

A1. "If I am performing an ultrasonography examination, I turn on the lights to report bad news"

( ) Totally disagree
( ) Partially disagree
( ) Neither agree nor disagree
( ) Partially agree
( ) Totally agree

A2."I ask pregnant women to sit down before delivering bad news"

( ) Totally disagree
( ) Partially disagree
( ) Neither agree nor disagree
( ) Partially agree
( ) Totally agree

A3. "I request that the pregnant patient get dressed before delivering bad news"

( ) Totally disagree
( ) Partially disagree
( ) Neither agree nor disagree
( ) Partially agree
( ) Totally agree

A4. "I take pregnant women to another room to report the bad news"

( ) Totally disagree
( ) Partially disagree
( ) Neither agree nor disagree
( ) Partially agree
( ) Totally agree

A5. "I consider the privacy of the environment in which the bad news is communicated to be important"

( ) Totally disagree
( ) Partially disagree
( ) Neither agree nor disagree
( ) Partially agree
( ) Totally agree

A6. "If a pregnant woman is alone, I ask if she wants to be accompanied by someone before communicating bad news"

( ) Totally disagree
( ) Partially disagree
( ) Neither agree nor disagree
( ) Partially agree
( ) Totally agree

A7. "When I break bad news, I first think about what I will say"

( ) Totally disagree
( ) Partially disagree
( ) Neither agree nor disagree
( ) Partially agree
( ) Totally agree

A8. "I feel comfortable breaking bad news"

( ) Totally disagree
( ) Partially disagree
( ) Neither agree nor disagree
( ) Partially agree
( ) Totally agree

A9. "I feel calm about breaking bad news"

( ) Totally disagree
( ) Partially disagree
( ) Neither agree nor disagree
( ) Partially agree
( ) Totally agree

A10. "I feel prepared to break bad news"

( ) Totally disagree
( ) Partially disagree
( ) Neither agree nor disagree
( ) Partially agree
( ) Totally agree

A11. "I have the ability to break bad news"

( ) Totally disagree
( ) Partially disagree
( ) Neither agree nor disagree
( ) Partially agree
( ) Totally agree

A12. "Before communicating bad news, I ask the patient what she knows about the situation"

( ) Totally disagree
( ) Partially disagree
( ) Neither agree nor disagree
( ) Partially agree
( ) Totally agree

A13. "I have the ability to deliver bad news about pregnancies"

( ) Totally disagree
( ) Partially disagree
( ) Neither agree nor disagree
( ) Partially agree
( ) Totally agree

A14. "I must know how much information the patient wants before communicating the information"

( ) Totally disagree
( ) Partially disagree
( ) Neither agree nor disagree
( ) Partially agree
( ) Totally agree

A15. "I am skilled in discussing the diagnosis"

( ) Totally disagree
( ) Partially disagree
( ) Neither agree nor disagree
( ) Partially agree
( ) Totally agree

A16. "I have the ability to discuss the prognosis"

( ) Totally disagree
( ) Partially disagree
( ) Neither agree nor disagree
( ) Partially agree
( ) Totally agree

A17. "I am skilled in talking about the end of pregnancy or the beginning of palliative care"

( ) Totally disagree
( ) Partially disagree
( ) Neither agree nor disagree
( ) Partially agree
( ) Totally agree

A18. "I am skilled in discussing issues related to the end of life"

( ) Totally disagree
( ) Partially disagree
( ) Neither agree nor disagree
( ) Partially agree
( ) Totally agree

A19. "I feel confident answering difficult questions asked by patients during the communication of bad news"

( ) Totally disagree
( ) Partially disagree
( ) Neither agree nor disagree
( ) Partially agree
( ) Totally agree

A20. "I had specific training on how to report bad news"

( ) Totally disagree
( ) Partially disagree
( ) Neither agree nor disagree
( ) Partially agree
( ) Totally agree

A21. "I consider the use of a strategy or protocol to communicate bad news important"

( ) Totally disagree
( ) Partially disagree
( ) Neither agree nor disagree
( ) Partially agree
( ) Totally agree

A22. "I have knowledge about the communication of bad news"

( ) Totally disagree
( ) Partially disagree
( ) Neither agree nor disagree
( ) Partially agree
( ) Totally agree

IF YOU HAVE EXPERIENCE WITH ANY PROTOCOL FOR DELIVERING BAD NEWS, ANSWER THE FOLLOWING QUESTIONS:

A23. "Although I know a communication protocol for bad news, I prefer to use my own clinical practices to communicate bad news"

( ) Totally disagree
( ) Partially disagree
( ) Neither agree nor disagree
( ) Partially agree
( ) Totally agree

A24. "I believe that the use of this protocol helps in clinical practice"

( ) Totally disagree
( ) Partially disagree
( ) Neither agree nor disagree
( ) Partially agree
( ) Totally agree

<u>Open-ended question</u>

Please briefly describe the protocol for breaking bad news that you know or use.

Supplementary Data S3. Fictitious Scenarios Based on Real Cases Used in the Role Playing Dramatization

Case 1

Scenarios

Pregnant woman, 30 years old, primigravida, with adequate prenatal follow-up, without previous intercurrences.

The physicians will perform the biopsy examination reading after investigation of suspicious breast nodules.

Role 1: Physician. You started prenatal care of the patient at 16 weeks of pregnancy. The patient's name is Amanda; she is 30 years old and is in her first pregnancy. At the first appointment, she brought the results of routine examinations that were performed before the sixth month of her pregnancy, as well as the results of the prenatal examinations that were requested; all results were normal. The prenatal care course was completed without adverse outcomes. Amanda is calm at all appointments, speaks little and always comes alone. Near 34 weeks of gestation, after seeing the results of her last prenatal routine examination, Amanda refers to the appearance of a nodule in the left breast. You examine Amanda and find the following:

Weight: 70 kg
Blood pressure: 110 × 70 mmHg
Fetal movements: present
Fetal heart frequency: 145 bpm
Symphysial fundal height: 31 cm

Breast: Presence of bulging in the medial superior quadrant of the left breast, hardened, adhered to deep planes, slightly mobile with ∼3.0 × 2.5 cm in size. Skin unchanged.

You become concerned about the characteristics of the nodule and request a breast ultrasonography to investigate.

After one week, the patient returns with the following result.

Breast ultrasonography: In the left breast in the medial superior quadrant, a nodular image with irregular contours, spicules, and a posterior acoustic shadow, measuring 1.5 × 1.0 cm, is noted. BI-RADS 4c.

Faced with this result, you request a biopsy as soon as possible and explain to the patient that this nodule worries you.

Today, at 37 weeks pregnancy, the patient returns to your office to obtain the results of the last ultrasonography and the core biopsy that was sent directly to you by the laboratory two days ago.

Fetal ultrasonography: 36 weeks, 2 days; cephalic presentation; amniotic fluid normal; estimated fetal weight 2943 g (40th percentile).

Core biopsy: Invasive carcinoma of no special type—WHO2012 (invasive ductal carcinoma).

You will have to talk to the patient about the diagnosis of breast cancer and schedule the birth.

Role 2: Patient. You' are a 30-year-old woman, working in office administration, and have been married for 3 years. Approximately one year ago, you started thinking about having a baby, and after a few months trying, you received the news that you were pregnant. You scheduled an appointment with an obstetric physician, whom you have visited annually to perform routine examinations, to begin prenatal care.

You always go to the physician's appointment because you go straight from work. Your husband works a lot, and your family lives in a rural city far from the city where you live.

In the city where you live, you can count on your husband's family and your aunt, who has adopted you since you came to the city. You do not like to disturb them, and because you feel very good, you prefer to do things yourself without disturbing them.

You are very happy because your first pregnancy occurred without any problems, unlike many friends who have had many bad symptoms as a result of pregnancy.

Since you became pregnant, you have read a lot about delivery and planning a normal delivery, which will be possible because everything is going well.

In the recent weeks, you felt a nodule in your breast. The physician requested some examinations to make sure it is nothing, and you did it all because you needed to take care of everything.

This week, you painted the room for the baby, and some friends had a surprise baby shower, and you can only imagine how it will be after the baby is born. You have many plans for your child and your family.

In a few days, you will have a 3D ultrasonography, and you will see the baby's face before the delivery; you are so excited.

Today, you go to a routine prenatal visit to see whether everything is going fine. As it is already the end of the pregnancy, your aunt asks to go with you because she wants to hear the baby's heart.

Role 3: Relative. You are a 57-year-old woman and have been retired for 4 years after discovering you had breast cancer. You did not marry and had no children, although you wanted them when you were younger. You adopted your sister's daughter when she came to the large city where you live after her wedding.

A few years ago, you were diagnosed with breast cancer and underwent a variety of treatments, including surgery, radiotherapy, and chemotherapy. The treatment was very difficult, but thank god, you are alive.

Supplementary Table S1. Effects of Training on the Distribution of Responses to the Affirmations on the Questionnaire

	Totally disagree, *N* (%)		Partially disagree, *N* (%)		Neither agree nor disagree, *N* (%)		Partially agree, *N* (%)		Totally agree, *N* (%)		
**Affirmations**	**S1**	**S2**	**S1**	**S2**	**S1**	**S2**	**S1**	**S2**	**S1**	**S2**	***p***
A1. "If I'm performing an ultrasonography examination, I turn on the lights to report bad news"	5 (5.7)	0 (00)	5 (5.7)	1 (2.6)	14 (16.1)	1 (2.6)	14 (16.1)	7 (17.9)	49 (56.3)	30 (76.9)	**0.010**
A2. "I ask pregnant women to sit down before delivering bad news"	0 (00)	1 (2.5)	4 (4.6)	1 (2.5)	7 (8.0)	1 (2.5)	17 (19.5)	8 (20)	59 (67.8)	29 (72.5)	0.552
A3. "I request that pregnant women get dressed before delivering bad news"	2 (2.3)	0 (00)	2 (2.3)	1 (2.5)	6 (6.9)	0 (00)	19 (21.8)	6 (15)	58 (66.7)	33 (82.5)	0.053
A4. "I take pregnant women to another room to report bad news"	2 (2.3)	1 (2.5)	5 (5.7)	3 (7.5)	30 (34.5)	8 (20)	28 (32.2)	17 (42.5)	22 (25.3)	11 (27.5)	0.404
A5. "I consider the privacy of the environment in which bad news is communicated to be important"	0 (00)	0 (00)	0 (00)	0 (00)	1 (1.1)	0 (00)	2 (2.3)	2 (5.1)	85 (96.6)	37 (94.9)	0.658
A6. "If a pregnant woman is alone, I ask whether she would like to be accompanied by someone before communicating bad news"	0 (00)	0 (00)	5 (5.7)	1 (2.5)	12 (13.8)	2 (5)	37 (42.5)	20 (50)	33 (37.9)	17 (42.5)	0.276
A7. "When I break bad news, I first think about what I will say"	1 (1.2)	0 (00)	0 (00)	0 (00)	3 (3.5)	0 (00)	8 (9.4)	8 (20)	73 (85.9)	32 (80)	0.485
A8. "I feel comfortable breaking bad news"	27 (31)	8 (20)	27 (31)	7 (17.5)	14 (16.1)	7 (17.5)	15 (17.2)	15 (37.5)	4 (4.6)	3 (7.5)	**0.013**
A9. "I feel calm about breaking bad news"	6 (7.0)	2 (5.0)	21 (24.4)	5 (12.5)	17 (19.8)	9 (22.5)	28 (32.6)	15 (37.5)	14 (16.3)	9 (22.5)	0.145
A10. "I feel prepared to break bad news"	5 (5.7)	0 (00)	24 (27.6)	7 (7.5)	18 (20.7)	2 (5.0)	24 (27.6)	23 (57.5)	16 (18.4)	12 (30)	**<0.001**
A11. "I have the ability to break bad news"	1 (1.1)	0 (00)	25 (28.4)	2 (5.0)	25 (28.4)	7 (17.5)	26 (29.5)	20 (50)	11 (12.5)	11 (27.5)	<**.001**
A12. "Before communicating bad news, I ask the patient what she knows about the situation"	0 (00)	0 (00)	1 (1.1)	1 (2.5)	3 (3.4)	2 (5.0)	16 (18)	10 (25)	69 (77.5)	27 (67.5)	0.221
A13. "I have ability to give space for pregnancy talks"	0 (00)	0 (00)	2 (2.3)	1 (2.5)	10 (11.4)	3 (7.5)	34 (38.6)	17 (42.5)	42 (47.7)	19 (47.5)	0.874
A14. "I try to know how much information the patient wants before communicating the bad news"	1 (1.1)	0 (00)	8 (9)	2 (5.1)	15 (16.9)	5 (12.8)	30 (33.7)	15 (38.5)	35 (39.3)	17 (43.6)	0.386
A15. "I am skilled in discussing the diagnosis"	1 (1.1)	0 (00)	12 (13.6)	1 (2.5)	15 (17)	6 (15)	37 (42)	19 (47.5)	23 (26)	14 (35)	0.076
A16. "I have the ability to discuss the prognosis"	4 (4.8)	0 (00)	14 (16.7)	3 (7.5)	20 (23.8)	7 (17.5)	36 (42.9)	23 (57.5)	10 (11.9)	7 (17.5)	**0.026**
A17. "I am skilled in talking about the end of pregnancy or the beginning of palliative care"	8 (9)	0 (00)	23 (25.8)	6 (15)	23 (25.8)	7 (17.5)	25 (28.1)	19 (47.5)	10 (11.2)	8 (20)	**0.003**
A18. "I am skilled in discussing issues related to the end of life"	7 (7.9)	1 (2.5)	30 (33.7)	6 (15)	18 (20.2)	9 (22.5)	25 (28.1)	17 (42.5)	9 (10.1)	7 (17.5)	**0.007**
A19. "I feel confident answering difficult questions asked by patients during the communication of bad news"	6 (6.7)	1 (2.5)	28 (31.5)	5 (12.55)	20 (22.5)	10 (25)	25 (28.1)	12 (32.5)	10 (11.2)	11 (27.5)	**0.004**

Bold values indicate values statistically significant differences between steps, $p < 0.05$.

You are very happy with your niece's pregnancy and are anxious to see the baby's face and who the baby will look like.

You do not like to visit the physician, but today, you are asking to go to the appointment with your niece to listen to the baby's heart and know how the niece's pregnancy is going. You are already imagining how it will be.

Case 2

Scenario

Pregnant woman, 34 years old, third gestation, in prenatal follow-up at the tertiary hospital due to fetal heart disease. The pregnant patient came in for ultrasonography evaluation and will be diagnosed with intrauterine fetal death.

Role 1: Physician. You are a physician in the department of obstetrics in the Hospital das Clínicas. As usual, you arrive on time and look at your schedule to see which patients will attend today and evaluate previous examinations.

The first patient who arrives is visiting the department due to a complex fetal heart disease.

After reading the last examinations performed by the cardiologic team, you call the patient to perform the ultrasonography. The patient responds to the call and appears with her husband. Today, she is 32 weeks pregnant and seems anxious about the test.

You start the examination and realize there are no fetal heartbeats; as you finish the examination, you think about how you are going to tell the family that the baby is dead.

Role 2: Patient. You are a 34-year-old woman, have been married for 10 years, and are pregnant for the third time; you are pregnant with a girl, and you already have two boys, one who is 7 years old and one who is 4 years old.

You were referred to the Hospital das Clínicas because based on prenatal scan, the baby was diagnosed with a minor heart problem. The physician said the baby will have surgery when she is born. You believe it will be a difficult time, but after that, you will take her home.

On the weekend that preceded the consultation, you, with the help of your family, threw a beautiful baby shower, where you received many gifts and organized things at home for the baby's arrival.

You feel that the baby is healthy because she moves a lot, and sometimes you wake up with the movements, and today was no different.

Role 3: Relative. You are a 34-year-old man, have been married for 10 years, and have two boys, one who is 7 years old and the other who is 4 years old; your wife is pregnant for the third time with a girl.

You were not expecting a pregnancy; it was a big surprise because your wife was using medication to avoid becoming pregnant. You are worried about the bills that you will have with another baby.

At 26 weeks of gestation, your wife was referred to the Hospital das Clínicas because the baby had a heart problem.

As there are many appointments that she has to attend, you cannot come to all of them because of work; your boss is not very understanding.

You tried to keep up with everything; your wife says that baby's minor heart problem can be corrected with a remedy or a surgery after she is born and that she grows well and is improving at each examination.

Today, you are happy to come to the appointment because you were released from work to accompany your wife to the hospital and you have not seen the face of your daughter for some time and have not listened to her heart.

Supplementary Data S4. Practical Training Feedback Questionnaire (Portuguese Language)

Questionário de Feedback do treinamento prático

Sua opinião é muito importante para nós, de forma anônima responda as seguintes questões:

1. De uma nota de 0 a 10:
    a. Quanto a comunicação de más notícias faz parte de sua rotina?
    b. Quanto sente dificuldade de transmitir essa notícia?
    c. Quanto acreditava estar preparado para tal situação?
    d. Quanto as situações encenadas simulam um caso real?
    e. Quanto de tempo em sua formação foi dedicado a atenção em comunicação?
    f. Quanto acha que o Role Play foi útil para sua formação?
2. O que podemos melhorar? Dê sua sugestão.

Supplementary Data S5. Feedback of practical training questionnaire (English translation)

Your opinion is very important to us, anonymously answer the following questions:

1. From a grade of 0 to 10:
    a. How much is communication a part of your life?

b. How difficult does it feel to break bad news?
c. How much time in your training or professional qualification was dedicated to communication attention?
d. How much did you think you were prepared for before the role play?
e. How much do you feel prepared to break bad news after role play?
f. How do the situations staged in role play simulate a real case?
g. How useful was the role play?

2. What can we improve? Give your suggestion.
